# Identification and characterization of a heme exporter from the MRP family in *Drosophila melanogaster*

**DOI:** 10.1186/s12915-022-01332-0

**Published:** 2022-06-02

**Authors:** Zhiqing Wang, Peng Zeng, Bing Zhou

**Affiliations:** 1 State Key Laboratory of Membrane Biology, School of Life Sciences, Tsinghua University, Beijing, 100084 China; 2 Institute for Immunology, Tsinghua-Peking Center for Life Sciences, School of Medicine, Tsinghua University, Beijing, 100084 China; 3 Shenzhen Institute of Synthetic Biology, Shenzhen Institutes of Advanced Technology, Chinese Academy of Sciences, Shenzhen, 518055 China

**Keywords:** ZnMP, MRP, Heme transport, *Drosophila*, Insects

## Abstract

**Background:**

The heme group constitutes a major functional form of iron, which plays vital roles in various biological processes including oxygen transport and mitochondrial respiration. Heme is an essential nutrient, but its pro-oxidant nature may have toxic cellular effects if present at high levels, and its synthesis is therefore tightly regulated. Deficiency and excess of heme both lead to pathological processes; however, our current understanding of metazoan heme transport is largely limited to work in mammals and the worm *Caenorhabditis elegans*, while functional analyses of heme transport in the genetically amenable *Drosophila melanogaster* and other arthropods have not been explored.

**Results:**

We implemented a functional screening in Schneider 2 (S2) cells to identify putative heme transporters of *D. melanogaster*. A few multidrug resistance-associated protein (MRP) members were found to be induced by hemin and/or involved in heme export. Between the two plasma membrane-resident heme exporters CG4562 and CG7627, the former is responsible for heme transit across the intestinal epithelium. CG4562 knockdown resulted in heme accumulation in the intestine and lethality that could be alleviated by heme synthesis inhibition, human MRP5 (hMRP5) expression, heme oxygenase (HO) expression, or zinc supplement. CG4562 is mainly expressed in the gastric caeca and the anterior part of the midgut, suggesting this is the major site of heme absorption. It thus appears that CG4562 is the functional counterpart of mammalian MRP5. Mutation analyses in the transmembrane and nucleotide binding domains of CG4562 characterized some potential binding sites and conservative ATP binding pockets for the heme transport process. Furthermore, some homologs in *Aedes aegypti*, including that of CG4562, have also been characterized as heme exporters.

**Conclusions:**

Together, our findings suggest a conserved heme homeostasis mechanism within insects, and between insects and mammals. We propose the fly model may be a good complement to the existing platforms of heme studies.

**Supplementary Information:**

The online version contains supplementary material available at 10.1186/s12915-022-01332-0.

## Background

Heme is an important molecule in all organisms and functions in oxygen delivery (globin), cellular energy generation (cytochromes and oxidoreductases), transcription regulation (Bach1) [1], circadian clock (nuclear hormone receptor, Rev-erb) [2, 3], microRNA processing (DGCR8) [4], xenobiotic detoxification (cytochrome P450s) [5], and so on [6, 7]. In *Drosophila melanogaster*, a hemoglobin gene (*glob1*) was identified displaying oxygen-binding ability and is mainly expressed in tracheal cells and fat body [8, 9]. Heme has also been shown to be an essential cofactor for the synthesis of the steroid hormone ecdysone [10]. The transcription factor Rev-erb is associated with heme ligand controlling metabolism and the clock in both mammals and *D. melanogaster* [11, 12].

In humans, heme originates from both de novo synthesis and external uptake from the diet, while in *Caenorhabditis elegans*, heme is solely taken up exogenously. The intracellular heme concentration is precisely and dynamically controlled by heme synthesis and degradation. Among all known cases, almost every organism shares a similar heme synthesis pathway named the Shemin pathway, except for some metazoans [13]. Using human genome as a reference, it has been found that homologs of all genes pertaining to the heme biosynthesis pathway exist in *D. melanogaster* and the blood-feeding dipteran *Aedes aegypti* [14]. In *D. melanogaster*, a single delta-aminolevulinate synthase (ALAS) gene (*dALAS*) has been found. *dALAS* mRNA is ubiquitously distributed and expression of the gene is inhibited by heme [15]. Notably, only one heme oxygenase (HO) has been identified in *D. melanogaster* and its catalytic efficiency is lower than that of mammalian HOs due to a quite different structure [16]. Ubiquitously knocking down *dHO* resulted in larva and pupa death [17].

Our understanding of heme trafficking in general is limited and arises mainly from mammalian and *C. elegans* studies. HCP1 was initially identified as a heme importer in the apical membrane of the intestine [18], while later it was proved to have a higher affinity for folate [19]. FLVCR1 is the cell surface receptor for feline leukemia virus subgroup C, and a highly homologous protein FLVCR2 was suggested to be an importer of extracellular heme [20]. In the presence of heme-binding protein, FLVCR1 could also export hemin [21, 22]. HRG-1 was first identified as a heme importer in *C. elegans* and subsequently proved to be conserved in mammals; the mammalian orthologue is predominantly expressed in the endosomal compartment trafficking heme into the cytosol [23]. Several papers reported that some proteins like ABCB10, ABCB6, and ABCG2 (similar to the product of *white* gene) may also be associated with heme transport processes [24–26].

Multidrug-resistant proteins (MRPs) belong to the ABCC family (ATP-binding-cassette subfamily C), which functions as primary-active transporters requiring ATP hydrolysis. MRP is so named because of its high expression level and ability to efflux numerous drugs, resulting in resistance of cancer cells to chemotherapy. Some physiological substrates of MRPs are determined, including folic acid, bilirubin, and oxidized or reduced glutathione [27]. MRP5 was first discovered as a heme exporter in the basolateral side of the intestine in the worm and was subsequently proved to be conserved in zebrafish and mammals [28]. Interestingly, MRP5 in the worm was later characterized as a vitamin B12 transporter important for the vitamin transmission from the mother to its offspring [29]. ABCC family in human consists of 12 members, which are characterized as transporters of different substrates often to cope with different physiological stresses. Functions of several MRP family members have been reported in *D. melanogaster*. *Drosophila* MRP4 (Mrp4) was identified in dealing with oxygen deprivation [30], and *Drosophila* MRP1 (Mrp) in modulating toxicity of methylmercury [31]. Cftr was studied in the fly gastrointestinal system to model the cystic fibrosis disease [32]. Applying functional RNAi screens, l(2)035659 was characterized as a bilirubin transporter in *D. melanogaster* [33].

*Aedes aegypti*, the yellow fever mosquito, is an insect capable of transmitting viruses whose infection could lead to human mortality [34]. Blood-meal is essential for mosquito growth and reproduction, which supplies two key nutrients, amino acids and iron. Previously, some putative heme importers or exporters have been identified in yellow fever mosquito through transcriptomic analyses. It is concluded that heme import may be controlled by a redundant mechanism [35]. Also, ABCB10 was identified in *Rhipicephalus* (Boophilus) *microplus*, which cannot self-synthesize heme, to function as the heme importer located in the hemosome of the midgut [36, 37].

*Drosophila melanogaster* offers a great insect model organism for researching many biological problems. Unfortunately, heme transport in *D. melanogaster* is virtually not explored [38]. Here, by taking advantage of the fluorescent heme analog ZnMP, we systematically screened an array of putative candidates for potential heme exporters. Several were identified and characterized with heme transport properties. Combining genetic, biochemical, and immunofluorescent methods, we revealed that *CG4562* works as a heme exporter in the intestine and plays a critical role in exporting heme to the body (presumably hemolymph), analogous to MRP5 in mammals. Furthermore, we found that the homolog of *CG4562* in yellow fever mosquito also functioned as a heme exporter. We hope that *D. melanogaster* will complement existing models of heme studies and improve our understanding of insect heme metabolism mechanisms, which may be of significance in pest control in the future.

## Results

### Screening the MRP family for heme transporters in S2 cells

**Fig. 1:**
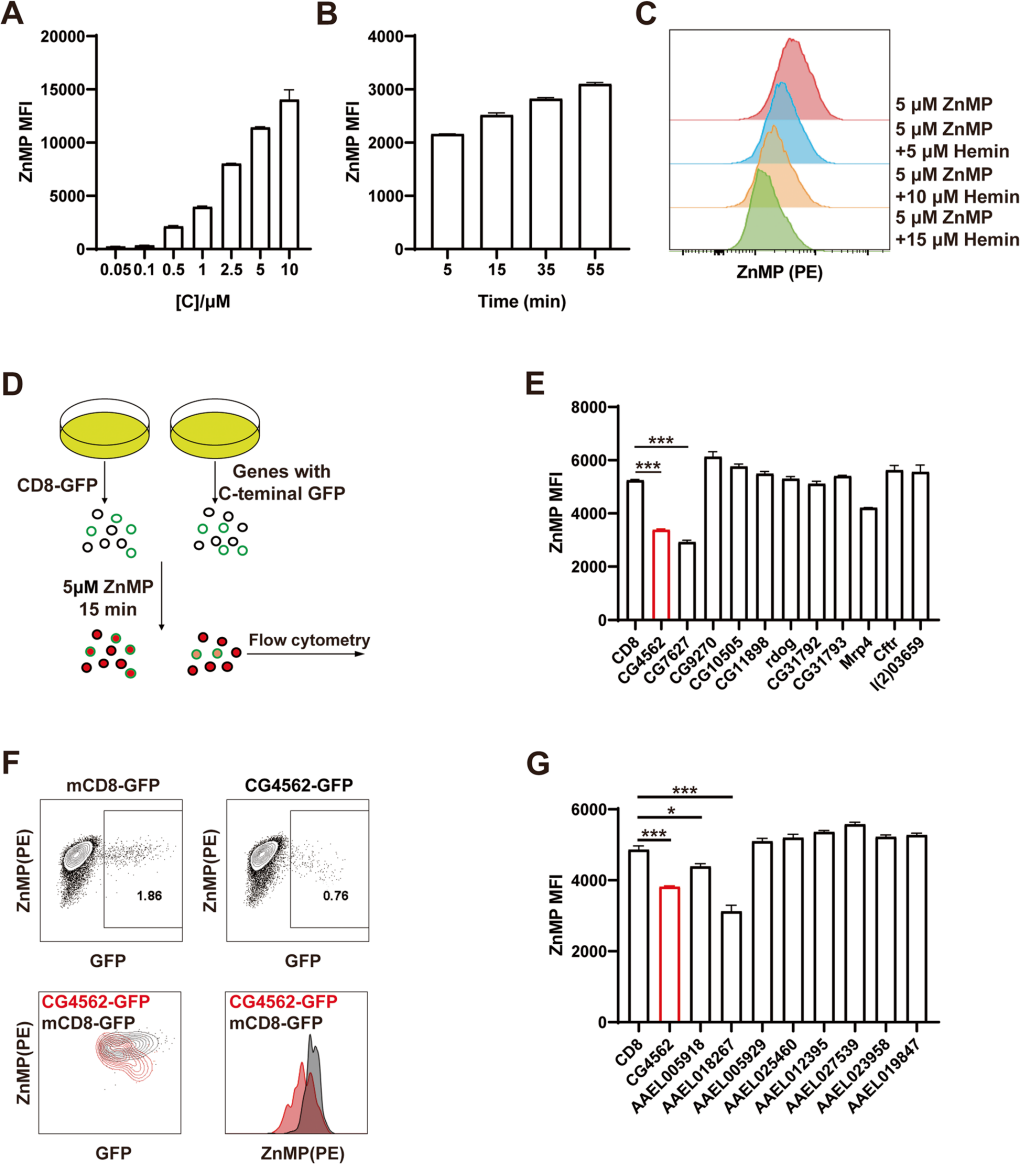
Identifying putative *Drosophila* heme transporters in S2 cells. **A, B** S2 cells were incubated with the fluorescent heme analog zinc-mesoporphyrin (ZnMP). ZnMP concentration effects on the fluorescence for 15 min treatment (**A**). The time course of the fluorescence with 5 μM ZnMP treatment (**B**). ZnMP detained in S2 cells after 15 min treatment was analyzed by flow cytometry. ZnMP MFI represents the mean fluorescence intensity of ZnMP in S2 cells. All values are available in Additional file 3: Fig. 1A and Fig. 1B. **C** ZnMP transport was competitively inhibited by heme in S2 cells. Various concentrations of heme were added together with ZnMP. ZnMP signal densities were analyzed in 15 min competition assay. **D** The scheme of screening putative heme transporters in S2 cells by 5 μM ZnMP fluorescence measurement. **E** Quantitative measurement of ZnMP fluorescence of different *Drosophila* MRP genes when overexpressed in S2 cells. All values are available in Additional file 3: Fig. 1E. **F** FACS analysis of the ZnMP signal density of mCD8-GFP (black) and CG4562-GFP (red) transfected S2 cells. Numbers in the left two figures indicated the frequency of transfected S2 cells in the gated region. **G** Quantitative measurement of ZnMP fluorescence when MRP4 homologous genes of *A. aegypti* were expressed in S2 cells. All values are available in Additional file 3: Fig. 1G. Data are presented as the mean ± SD by two-tailed unpaired Student’s *t* test. *p < 0.05, **p < 0.01; ***p < 0.001. Experiments were repeated at least two times, and data from only one representative experiment were shown

The importance of heme physiology was appreciated years ago. In *D. melanogaster*, heme synthesis is conserved compared to the mammals and shared all the heme synthesis proteins (Additional file 1: Fig. S1A). Ubiquitous knockdown of the first rate-limiting heme synthesis enzyme dALAS (CG3017) resulted in lethality, which was rescuable by exogenous substrate ALA (Additional file 1: Fig. S1B). This suggests that endogenous heme synthesis is essential for *D. melanogaster* survival, alike to that in mammals and most other organisms but different from the worm. Noteworthy is that a previous study also identified dALAS required for apical transcellular barrier formation in the skin of *D. melanogaster* larva [39]. To study heme transport in *D. melanogaster*, a convenient screen system was established to detect heme transport in S2 cells. Zinc mesoporphyrin (ZnMP), a fluorescent heme analog, can be easily monitored by microscopy and flow cytometry [35, 40]. With increasing concentrations and time, the fluorescence signals grew simultaneously, indicating that ZnMP absorption is dose- and time-dependent in S2 cells (Fig. 1A, B). Uptake of ZnMP indeed happened as shown in the movie files (Additional file 2: movie S1 and movie S2). ZnMP fluorescence decreased following heme treatment, and the signal was lower with higher levels of heme (Fig. 1C). These data indicated that quantitative analysis of ZnMP fluorescence in cells by flow cytometry offered us a useful tool to identify heme transporters in S2 cells.

MRP5, a short MRP belonging to the ABCC family, was uncovered to function as a heme transporter so we wondered whether there could be any MRP-like protein taking part in heme homeostasis in *D. melanogaster*. A BLASTP search using the protein sequences of 12 human ABCC proteins, also named multidrug-resistant proteins (MRPs), revealed that there exist 14 members of the ABCC family in *D. melanogaster* (Additional file 1: Fig. S1C). Based on the number of predicted transmembrane domains (TMDs), the ABCC family consists of two groups—the long protein with three TMDs and the short proteins containing two. Sulfonylurea receptor (SUR), one of the long MRPs members, is an ATP-dependent potassium channel regulator and duplicates to SUR1 and SUR2 in mammals [41]. There is only one homolog of SUR1/SUR2 in *D. melanogaster* named Sur [42], and CG7806 is closely homologous to MRP7. Previously, CG6214 (Mrp) in the flies was identified as the close homolog of human MRP1 [43]. MRP1 and MRP7 are also the long MRPs reported facilitating some drugs out of cancer cells [41]. The other eleven members of the MRP family are short MRPs closely related to MRP5, but they seem to be at least as close to MRP4 based on the phylogenetic tree (Additional file 1: Fig. S1C). It is unclear whether the eleven short MRPs are related to heme homeostasis, so we implemented an ZnMP transporting assay to check their heme transporting activity in vitro. Considering that these eleven members are relatively similar, we GFP-tagged them at the C-terminal and over-expressed them in S2 cells. CD8-GFP was utilized as a control (Fig. 1D). The ZnMP fluorescence of GFP-positive cells was detected by flow cytometry after ZnMP incubation and then the signal differences between CD8-GFP and MRP-GFP proteins were analyzed (Fig. 1E). Among the eleven genes tested, ZnMP fluorescence signal significantly decreased upon the expression of two genes, indicative of direct heme export. Noteworthy is that the CG7627 exporting activity was slightly more potent than that of CG4562 (Fig. 1E). For reasons that will become clear further below, we focused our analysis on CG4562. A different assay using FACS analysis corroborated our initial observation that this transporter could export heme from S2 cells; the ZnMP signal after transfection of the CG4562 was much lower than the control (Fig. 1F). Additional movie files showed ZnMP exported by CG4562-GFP compared to CD8-GFP (Additional file 2: movie S3 and movie S4).

Despite that a transcriptional analysis has been performed in *A. aegypti* [35, 44, 45], there is no functional study of heme export in mosquito species. As both the model organism *D. melanogaster* and the mosquito species *A. aegypti* belong to the *Diptera* and are therefore related in evolutionary terms, we wondered whether the heme transporters identified in *D. melanogaster* are conserved in *A. aegypti*. BLASTP searches using the protein sequences of the eleven *D. melanogaster* MRP4 homologous proteins showed that the yellow fever mosquito genome possesses eight proteins of this family (Additional file 1: Fig. S1D). Similarly, we repeated the ZnMP screen in S2 cells expressing eight *A. aegypti* genes. The results revealed that two genes had potential heme export activity (Fig. 1G). Surprisingly, although AAEL012395 was proposed to be a heme exporter in *A. aegypti* and was upregulated by heme in the midgut [35], we did not find any obvious alteration in heme effluxing activity compared to S2 cells expressing CD8-GFP. Nevertheless, two new genes *AAEL005918* and *AAEL018267* displayed decreased ZnMP signals, with AAEL018267 as the more potent transporter between the two (Fig. 1G). Additionally, the expression level of AAEL005918 increased under exogenous heme stimulation (Additional file 1: Fig. S2C). We proposed AAEL005918 and AAEL018267 as potential heme exporters in the *A. aegypti*. Collectively, two MRP4 homologs in *D. melanogaster* and *A. aegypti* showed potential heme export activity in S2 cells based on ZnMP assays.

### Knockdown of heme-responsive CG4562 in the whole body or gut resulted in a growth defect

**Fig. 2:**
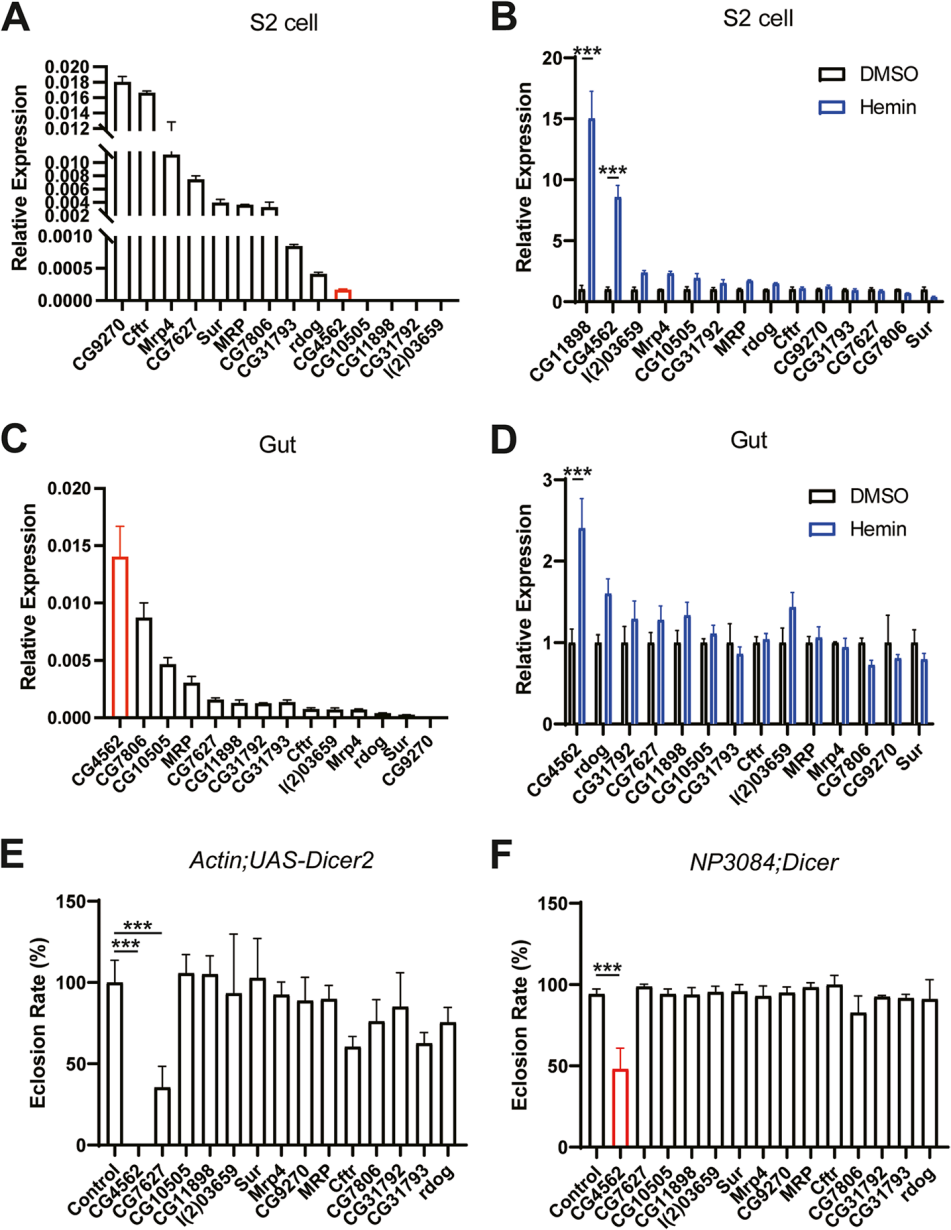
*CG4562* is heme-inducible and knocking down in the whole body and the gut resulted in growth defects. **A** Endogenous expressions of the MRP genes in S2 cell without hemin treatment. Expressions quantified by real-time relative RT-PCR normalized to *rp49*. All values are available in Additional file 3: Fig. 2A. **B** Transcription level changes of the MRP genes induced by 100 μM heme in S2 cell. Expressions of MRP genes after 4 h of heme induction were quantified by qPCR normalized by *rp49*. Expression values are relative to those with DMSO treatment. All values are available in Additional file 3: Fig. 2B. **C** Endogenous expressions of the MRP genes in the gut of the 3rd instar larva quantified by relative PCR normalized to *rp49*. All values are available in Additional file 3: Fig. 2C. **D** Relative transcription level changes of the MRP genes in the gut when supplementing 1 mM hemin in the food. Expressions quantified by relative PCR normalized to *rp49*. Expression is relative to that with DMSO treatment. All values are available in Additional file 3: Fig. 2D. **E** Phenotypic analyses of the MRP genes after knocking down in the whole body by *Actin-GAL4* directed RNAi. Eclosion rate normalized to the control. All values are available in Additional file 3: Fig. 2E. **F** Phenotypic analyses of the MRP genes after knocking down in the gut by *NP3084-GAL4*-directed RNAi. All values are available in Additional file 3: Fig. 2F. Data are presented as means ± SD by two-tailed unpaired Student’s *t* test. *p < 0.05, **p < 0.01; ***p < 0.001. Experiments were repeated at least two times, and data from only one representative experiment were shown. The RNAi lines and their phenotype summary of this screening were attached in Additional file 1: Table S1

After examining the heme efflux activity of various MRP members, our assays identified two proteins that could potentially transport ZnMP out of cells. We next analyzed the expression of these genes and their responses to hemin in S2 cells and the gut. One of them, *CG9270*, is endogenously expressed the highest in S2 cells while *l(2)03659* expressed the lowest (Fig. 2A). The endogenous expression levels of these genes in S2 cells differed by about ten thousand times. We then sought to determine whether these MRP genes are regulated by exogenous hemin. Under different heme-exposed concentrations, *CG11898* and *CG4562* of the fourteen MRP genes were significantly more differentially expressed, although to a much less extent a few others also might be slightly upregulated (Fig. 2B, Additional file 1: Figure S2B). The robust responsiveness of *CG4562* and *CG11898* to hemin suggested that these two genes may relate to heme metabolism. Since it is known that besides as a nutrient, heme is also a toxic molecule, we figured that when absorption or synthesis of excess heme occurred, a precise regulation to balance the heme concentration should be evolved in the gut. One way of regulation is to efflux the heme. To figure out which gene is responsible for this heme transport process, we first evaluated the endogenous MRP expression levels in the gut. Our data showed that *CG4562* has the highest expression in the gut, consistent with the information reported in the FlyBase database (Fig. 2C, Additional file 1: Fig. S3B). *CG4562* expression robustly responded to exogenous hemin in vivo; rearing flies in hemin food upregulated *CG4562* expression in the gut, while the other MRP family members did not manifest much change (Fig. 2D). These initial pieces of evidence implied that *CG4562* may function in the flies’ gut in response to exogenous hemin. We next asked whether any of these MRP genes has any physiological function in the flies. To this end, we first ubiquitously knocked down the MRP members in the whole body by *Actin-GAL4* (Fig. 2E). Of the eleven MRP4 homologs, only *CG4562* and *CG7627* RNAi exhibited growth lethality, corresponding interestingly to the two candidates identified from the prior export screen experiment. We then characterized the potential function of these putative heme transporters in the gut. Knocking down in the gut by *NP3084-GAL4* showed that only *CG4562* produced a decrease of survival or eclosion (Fig. 2F). Notably, *CG7627* knockdown in the gut did not manifest obvious defects, suggesting that CG7627 may primarily function in some places outside the gut. Together, these data pinpointed *CG4562* as the physiologically vital and potential heme exporter in the gut.

### Other potential heme transporters (non-MRP transporters) in S2 cells and their physiological functions in the whole body and the gut

**Fig. 3:**
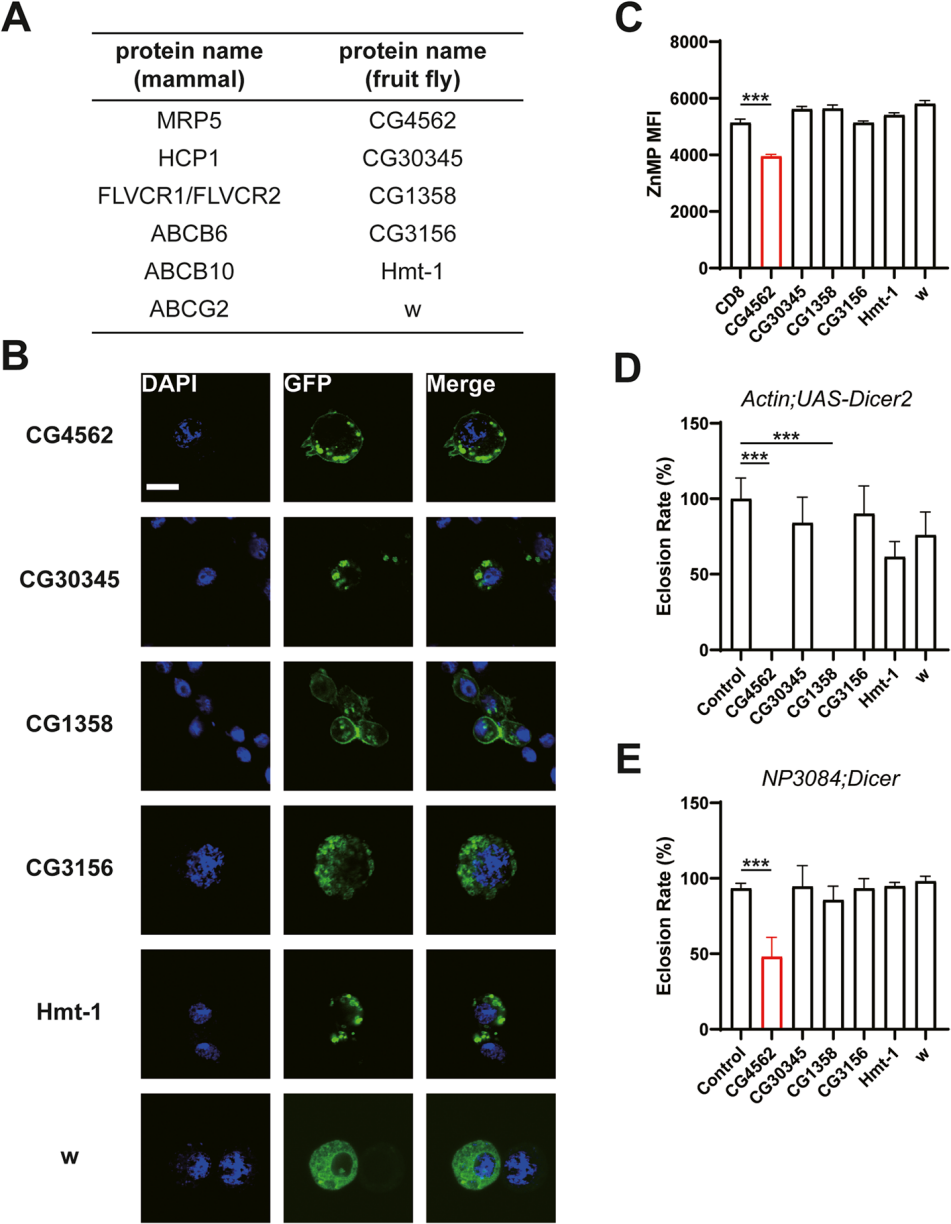
Characterization of some other potential *Drosophila* heme transporters (non-MRP transporters). **A** A list of other potential heme transporters in *Drosophila*. **B** Localization of these potential heme transporters when overexpressed in fusion with GFP in S2 cells. Scale bar = 10 nm. **C** Quantitative measurement of ZnMP fluorescence of these potential *Drosophila* heme transporters when overexpressed in S2 cells. All values are available in Additional file 3: Fig. 3C. **D, E** Phenotypic analyses of these potential heme transporters when knocking down in the whole body (*Actin-GAL4*) (**D**) and the gut (*NP3084-GAL4*) (**E**)*.* Eclosion rate normalized to the control. All values are available in Additional file 3: Fig. 3D and Fig. 3E. Data are presented as means ± SD by two-tailed unpaired Student’s *t* test. *p < 0.05, **p < 0.01; ***p < 0.001. The experiments were repeated at least two times, and only one representative one was shown. The RNAi lines and their phenotype summary of this screening were attached in Additional file 1: Table S1

In a similar approach, five other potential non-MRP *D. melanogaster* heme transporters homologous to known ones were identified, and their heme transporting activities were subsequently assayed in S2 cells (Fig. 3A). HRG-1 was first identified in *C. elegans* functioning as an importer and located in the plasma membrane as well as the vesicle. Through protein blast, there is no obvious HRG-1 homolog in *D. melanogaster*, so whether there exists a functional HRG-1 counterpart in *D. melanogaster* is not known. Only CG1358 was found sharing homology with the FLVCR heme transporter located on the plasma membrane (Fig. 3B). A *GAL4* inserted into the first intron of *CG1358*, when reported by RFP, indicates that *CG1358* is expressed in the brain [46] and RNA interference of this gene in circadian clock neurons disrupted rhythmic behavior in *D. melanogaster* [47]. The other four potential *D. melanogaster* heme transporters homologous respectively to HCP1, ABCB6, ABCB10, and ABCG2 are encoded by *CG30345*, *Hmt-1*, *CG3156*, and *white* (Fig. 3A). Notably, *white* encodes a transporter moving pigment precursors and involves in zinc storage in *D. melanogaster* [48], while *Hmt-1* functions in heavy metal detoxification [49]. We undertook the ZnMP screen as described earlier to analyze these five genes’ heme transport activities. To our surprise, the ZnMP fluorescence signal remained little changed when these genes were overexpressed (Fig. 3C). Localization studies revealed that unlike CG1358, which is located on the plasma membrane, the other four genes’ products did not exhibit obvious plasma membrane signals (Fig. 3B). Their physiological indispensability in flies was checked by knockdown studies in vivo. Notably, ubiquitous knockdown of *CG1358* by *Actin-GAL4* resulted in lethality, but there was no obvious survival phenotype when knocking it down in the gut (Fig. 3D). Given that *CG1358* is expressed highest in the brain, we then tested knockdown in the brain with *Elav-GAL4* (Additional file 1: Fig S2D). The phenotype with brain knockdown is similar to that seen with *Actin-GAL4*. Ubiquitous and gut-specific knockdown of the other four potential heme transporters did not present any severe phenotype (Fig. 3E). Collectively, none of these potential heme transporters functions similarly to CG4562 in the intestine. CG4562 seems to be the primary plasma membrane-resident heme exporter in the gut.

### CG4562 is highly expressed in the gut and localized to the surface of the gut

**Fig. 4:**
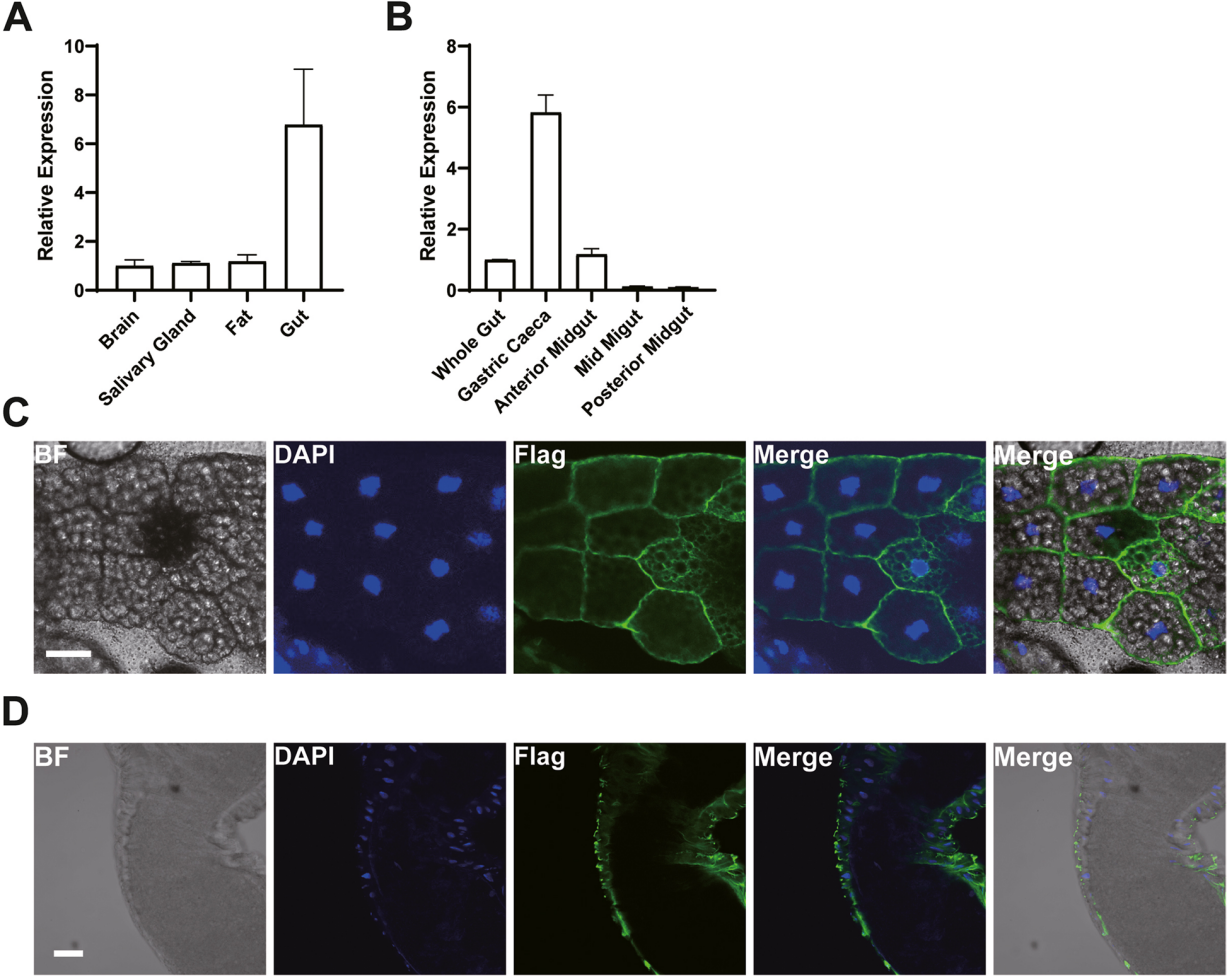
CG4562 expression is enriched in the intestine and at least partially localized to the basolateral side of the gut. **A** Relative *CG4562* expressions in different tissues to that of the brain as quantified by relative PCR normalized to *rp49*. All values are available in Additional file 3: Fig. 4A. **B** Relative *CG4562* expressions in different parts of the 3rd instar larva gut relative to that of the midgut as quantified by relative PCR normalized to *rp49*. The anterior midgut excluded the gastric caeca part. All values are available in Additional file 3: Fig. 4B. **C, D** Localization of over-expressed CG4562-Flag. In the fat body, scale bar = 30 μm (**C**) and in the gut (**D**), scale bar = 60 μm. Data are presented as means ± SD. The experiments were repeated at least two times, and only one representative one was shown

Expression analyses of *CG4562* revealed that *CG4562* is mainly expressed in the gut and much weaker in other tissues (Fig. 4A). More refined expression studies showed that *CG4562* is mainly expressed in the gastric caeca and the anterior midgut of the intestine, while rarely expressed in the middle and posterior midguts (Fig. 4B). In theory, the heme in the gut could be detoxified by export into the lumen or to the hemolymph for systemic use. If *CG4562* functions in heme uptake in vivo, we expect some expression of *CG4562* in the plasma membrane at the basolateral side of the gut. To identify the subcellular localization of CG4562, we expressed GFP-tagged CG4562 in S2 cells. The majority of fluorescence signals appeared to be on the plasma membrane (Fig. S2). Likewise, *D. melanogaster* close homolog of MRP4 shared a similar location in S2 cells (Additional file 1: Fig. S2A). Introducing Flag-tagged *CG4562* to *D. melanogaster* revealed plasma membrane localization in the fat body (Fig. 4C). Careful examination found the uneven distribution of *CG4562* on the basolateral membrane of the enterocytes (Fig. 4D), with also expression on the luminal side. Because this is tagged overexpression, we cannot be sure whether all the expression pattern reflects the physiological in vivo scenario. Nevertheless, this distribution pattern is consistent with the notion that *CG4562* executes the function of heme efflux from the gut at least partially to the body for systemic use.

### Knocking down CG4562 in the gut led to corresponding heme accumulation and lethality rescuable by SA or hMRP5 expression

**Fig. 5:**
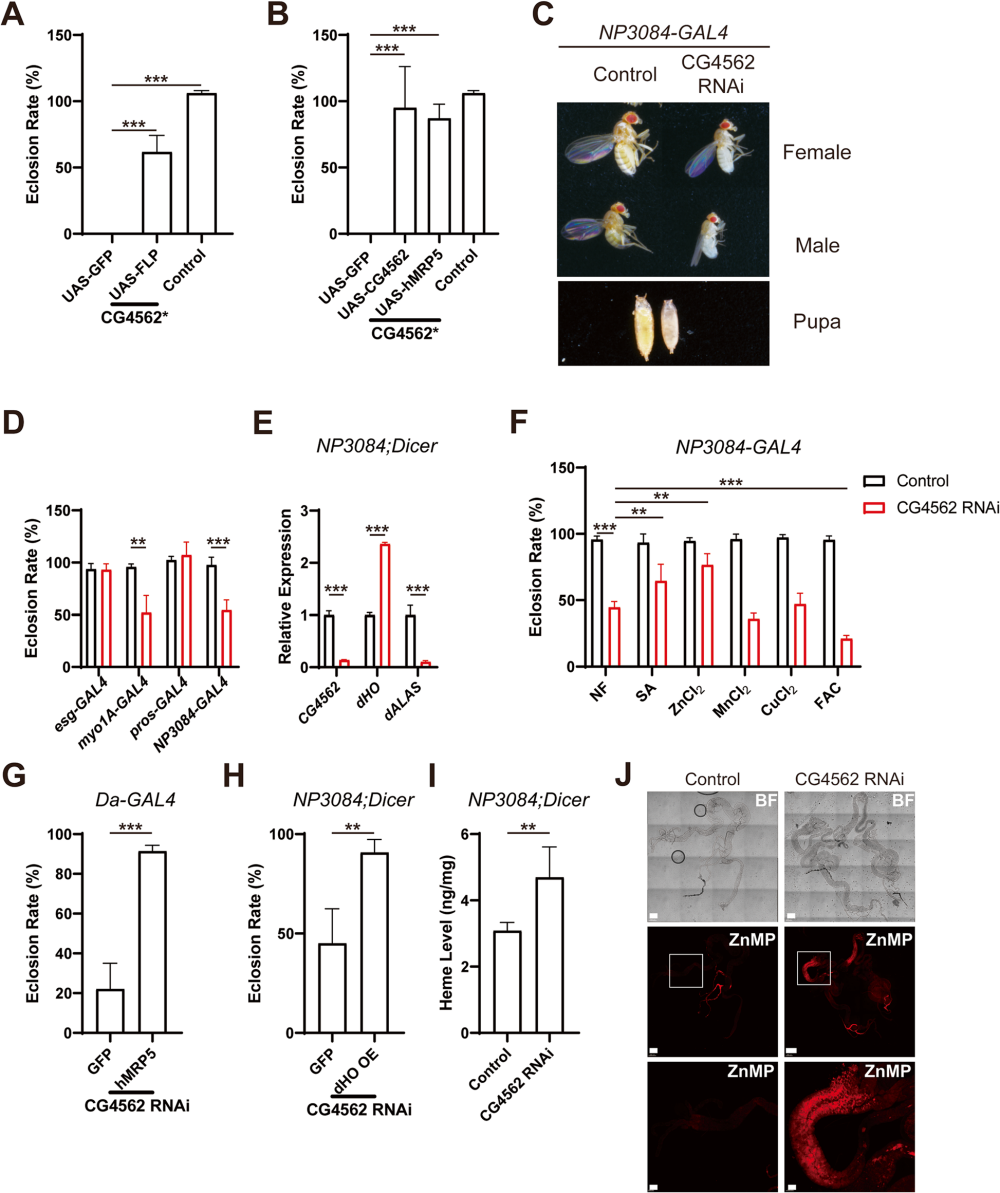
Knocking down *CG4562* in the gut led to an eclosion defect rescuable by SA, zinc, and expression of hMRP5 or HO. **A, B** The CG4562* mutant lethality could be rescued by insertion excision after FLP introduction. CG4562* is a mutation caused by the insertion of a Flippase-excisable *GAL4* cassette into the first intron of *CG4562*. When crossing with Flippase flies (**A**) or UAS-hMRP5 and UAS-CG4562 flies (**B**), lethality was reversed. All values are available in Additional file 3: Fig. 5A and Fig. 5B. **C** A picture showing the flies and pupae after *CG4562* knockdown in the gut by *NP3084-GAL4*. **D** Eclosion rates after knocking down *CG4562* by different gut Gal4s. *esg-GAL4* expresses in the ISCs, *myo1A-GAL4* expresses in the EC cells, *pros-GAL4* expresses in the EE cells, and the *NP3084-GAL4* expresses in the whole midgut. All values are available in Additional file 3: Fig. 5D. **E** Expression levels of several heme-related genes after knocking down *CG4562* in the 3rd instar larva gut by *NP3084-GAL4*. Quantification was by relative PCR normalized to *rp49*. All values are available in Additional file 3: Fig. 5E. **F** Chemical rescue of *CG4562* knockdown in the gut by *NP3084-GAL4*. Chemical concentrations used in this experiment: SA, 0.1 mg/ml; ZnCl_2_, 2 mM; MnCl_2_, 2 mM; CuCl_2_, 0.25 mM; FAC, 2 mM. All values are available in Additional file 3: Fig. 5F. **G** hMRP5 expression rescued *CG4562* knockdown. Expression directed by the whole-body *Da-GAL4* driver. UAS-GFP was used as the control. All values are available in Additional file 3: Fig. 5G. **H** dHO OE rescued *CG4562* knockdown in the gut by *NP3084-GAL4*. All values are available in Additional file 3: Fig. 5H. **I** Intestinal heme level was increased after knocking down *CG4562* in the gut by *NP3084-GAL4*. All values are available in Additional file 3: Fig. 5I. **J** Distribution of the heme analog (ZnMP) when knocking down *CG4562* in the gut by *NP3084-GAL4*. Scale bar for the two-upper pictures: 300 μm. The below panel is zoomed-in pictures for the white boxed regions. Scale bar for the below panel: 100 μm. Data are presented as means ± SD by two-tailed unpaired Student’s *t* test except for **F**, which is by one-way ANOVA test. *p < 0.05, **p < 0.01; ***p < 0.001. Experiments were repeated at least two times, and data from only one representative experiment were shown

One mutant of *CG4562* is available in the fly database, generated by a Flippase-excisable *GAL4* insertion cassette [46] in the first intron of the gene. Homozygotes of this mutation are lethal. This lethality could be reversed by Flippase-catalyzed excision and rescued by a single UAS construct containing *CG4562* cDNA (Fig. 5A), excluding the possibility that the lethality arises from other irrelevant mutations. In addition, UAS-hMRP5 was able to rescue the mutant phenotype (Fig. 5B), indicating *CG4562* could be functionally substituted by *hMRP5*.

We wondered why the loss of *CG4562* is lethal. According to MRP5 studies in mammals, we suspected CG4562 might efflux heme from the gut and play a role in heme absorption. If this is the case, the lethality should come from heme deficiency in the body or alternatively heme accumulation somewhere such as in the gut. To investigate these possibilities, we knocked down this gene with different tissue-specific GAL4 drivers (Table 1). *CG4562* knockdown mediated by RNAi in the whole body, directed with *Da-GAL4* or *Actin-GAL4*, was lethal, mimicking the mutant phenotype just described. Meanwhile, knockdown in the gut by *NP3084-GAL4* showed a reduced eclosion rate, growth retardation of 5–6 days, smaller pupa, and adult phenotypes (Fig. 5C). In comparison, knocking down this gene in many other tissues produced no obvious phenotypes (Table 1), indicating the gut as one of the principal functional areas of *CG456*2. To further delineate which kind of gut cells is involved in CG4562 function, we exploited different gut GAL4s to knock down *CG4562* in discrete cells (Additional file 1: Fig. S3A). Knocking down by *esg-GAL4* and *pros-GAL4* produced no obvious phenotypes, while *myo1A-GAL4* directed CG4562 knockdown caused a similar phenotype as that by *NP3084-GAL4* (Fig. 5D), supporting that *CG4562* is indispensable in the enterocyte cells of the midgut rather than intestinal stem cells or enteroendocrine cells.

**Table 1:** Phenotypic analyses of knocking-down CG4562 in different tissues by different GAL4 lines

GAL4	Expression tissue	Knockdown phenotype (THU1025, 4562R-1)
*Actin/Cyo*	Whole body	Lethal
*Daughterless*	Whole body	Lethal at the 2^nd^ instar larva
*NP3084*	Midgut	Growth delay for 5–6 days and smaller flies
*A9*	Wing	Normal
*GMR*	Eye	Normal
*Eyeless*	Eye	Normal
*Lsp*	Fat body	Normal
*Muscle*	Most in muscle	Normal
*Elav*	Neuron	Normal
*Mhc*	Muscle	Normal
*esg*	ISC cells	Normal
*pros*	EE cells	Normal
*myo1A*	EC cells	Growth delay for 5–6 days and smaller flies, same as NP3084

One possible cause underlying the lethality of *CG4562* knockdown may arise from heme accumulation in the gut. To explore this possibility, we first tested the expression of some heme-sensitive gene markers. *CG4562* knockdown in the gut upregulated *D. melanogaster* heme oxygenase gene and strongly repressed dALAS expression (Fig. 5E), consistent with the expectation of heme elevation in the gut. This means that CG4562 may normally efflux heme from the gut. If this is indeed the case, we expect blocking endogenous heme synthesis could rescue the phenotype. SA (succinyl-acetone) is a heme synthesis inhibitor targeting ALAD, the third enzyme of the Shemin pathway. As expected, SA well rescued the lethality as a result of the *CG4562* knockdown. Interestingly, zinc supplementation also rescued but FAC aggravated the phenotype (Fig. 5F). This is likely due to that heme requires iron incorporation, and there may exist an antagonism between zinc and iron, which is also observed under some other scenarios [50–52].

If CG4562 functions analogously as hMRP5 and export heme, we anticipate that besides hMRP5, dHO could also relieve heme toxicity in the gut and rescue *CG4562* RNAi (knockdown) phenotypes. We have mentioned that ubiquitous hMRP5 expression rescued *CG4562* RNAi in the whole body (Fig. 5G). Indeed, dHO overexpression could also well rescue the *CG4562* RNAi phenotype (Fig. 5H).

To obtain more direct pieces of evidence affirming the heme-effluxing function of CG4562, we monitored whether heme is accumulated after its disruption. Heme accumulation after *CG4562* knockdown in the gut was proved directly by UPLC (Fig. 5I). In addition, we utilized ZnMP to trace heme movement. Compared to normal larvae, *CG4562* RNAi larvae fed with ZnMP diet displayed an obvious fluorescent signal in the anterior midgut while there was no difference in other parts of the gut (Fig. 5J). Altogether, we conclude that *CG4562* functions as a heme exporter in the gut, analogous to MRP5. In that sense, CG4562 is dMRP5. While our phylogenic tree displays a closer relationship between CG4562 and MRP4, different analyses may produce slightly different results. The overall sequence similarity between CG4562 and MRP4 is rather comparable to that between CG4562 and MRP5.

### CG4562/dMRP5 transport mechanism in S2 cells

**Fig. 6:**
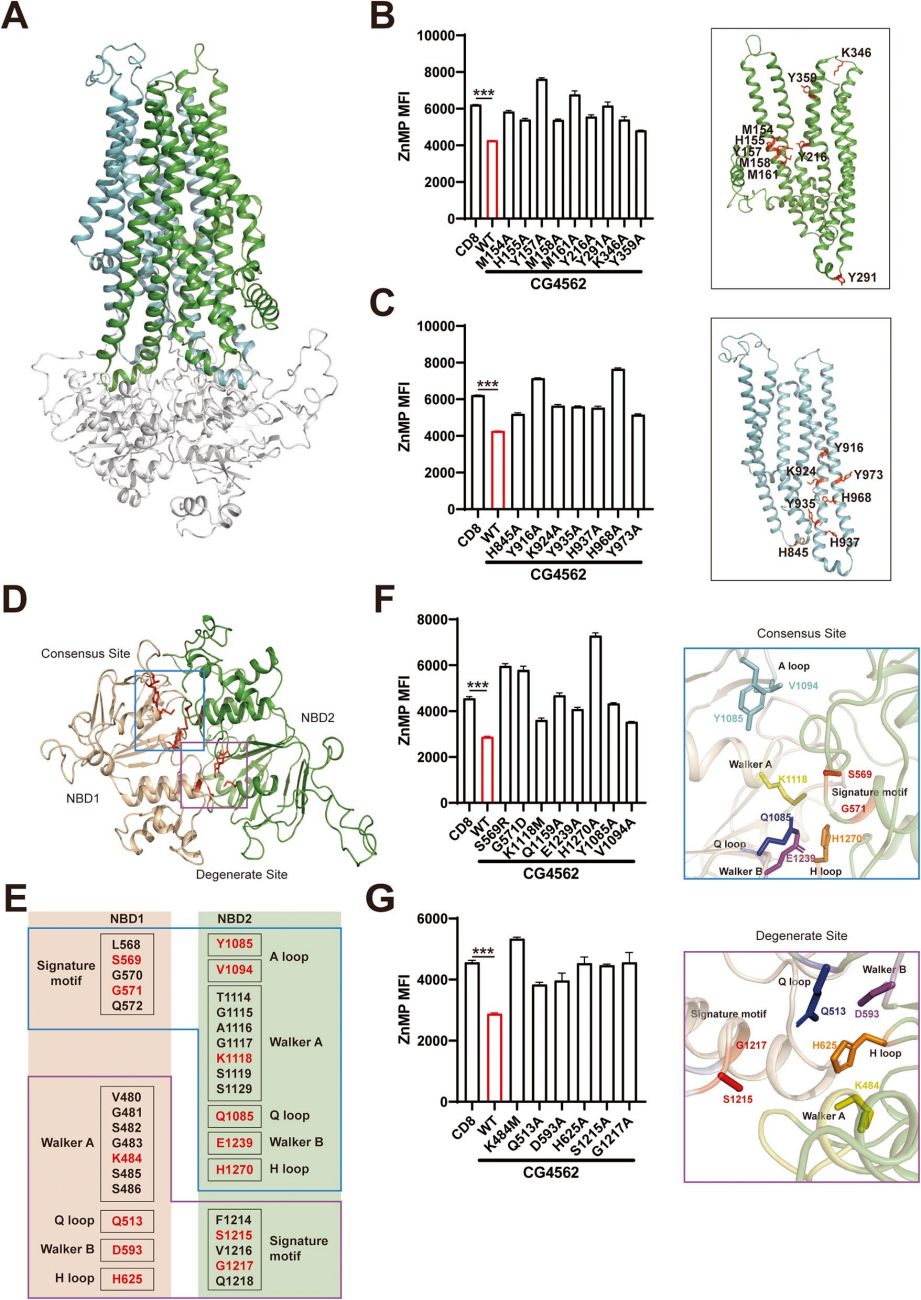
Critical sites for CG4562/dMRP5 heme transport. **A** CG4562/dMRP5 structure was homology modeled based on human CFTR. The transmembrane domain TMD1 (green), TMD2 (cyan), and NBD domains (gray) are marked respectively. The side chains of functional residues in TMD1 and TMD2 are labeled in red. **B, C** Potential heme-binding amino acids, of which side chains are inward were selected and mutated into alanine. ZnMP retention signals were analyzed with S2 cells expressing CG4562/dMRP5 TMD1 (**B**) and TMD2 (**C**) mutants. All values are available in Additional file 3: Fig. 6B and Fig. 6C. **D** The nucleotide binding domains of CG4562/dMRP5. NBD1 (wheat) and NBD2 (green) form two ATP binding sites consisted of the consensus site (blue box) and the degenerate site (purple box). **E** Detailed features of the consensus and degenerate sites are shown and key amino acid residues are highlighted (red). **F**, **G** Key amino acids from the consensus (**F**) and degenerate (**G**) sites were mutated. ZnMP signal density was analyzed. Zoom-in views of the consensus and degenerate sites are on the right side. All values are available in Additional file 3: Fig. 6F and Fig. 6G. Data are presented as means ± SD by two-tailed unpaired Student’s *t* test. *p < 0.05, **p < 0.01; ***p < 0.001. Experiments were repeated at least two times, and data from only one representative experiment were shown

After identifying CG4562/dMRP5 as the essential heme exporter in *D. melanogaster* gut, some biochemical characterization was subsequently carried out of the gene. Heme transporting by CG4562/dMRP5 is heme concentration-dependent. As ZnMP concentrations increased, the differences to the CD8-GFP control widened (Additional file 1: Fig. S4A). The mechanism of how MRP5 transporting heme has yet to be elucidated. For one, the crystal structure information related to heme transport remained obscure. Primary structure alignment of CG4562/dMRP5 to several human MRP family members highlights conservation of two transmembrane domains (TMD) and two nucleotide-binding domains (NBD), shared by MRP4, MRP5, MRP8, and MRP9 (Fig. 6A).

By homology model construction to CFTR [53], we obtained a preliminary structure of CG4562/dMRP5. Like other typical ABCC family members, CG4562/dMRP5 contains an N-terminal lasso motif, the first transmembrane domain (TMD1), the first nucleotide-binding domain (NBD1), the second transmembrane domain (TMD2), and the second nucleotide-binding domain (NBD2) (Additional file 1: Fig. S4B).

The cavity formed by TMD1 and TMD2 is assumed to bind and transport heme. Based on a previous study, there exist several potential heme-binding amino acids such as H, M, C, Y, and K [54]. Among them, histidine is the dominant residue found in the heme-binding proteins. Furthermore, the side-chain orientation of the amino acid in the heme-binding pocket is also vital for its association with heme. Thus, we selected putative heme-binding residues located in the TMDs and with the inward side chain and constructed expression plasmids of all these CG4562/dMRP5 variants.

These CG4562/dMRP5 mutants were subject to heme-transporting assays after introducing into S2 cells. Nonfunctional sites of TMD domains had been ruled out (Additional file 1: Fig. S4E); some mutations appeared to confer higher activities. We have found different patterns of heme-associated residues in the TMD1 and TMD2 domains. In TMD1, M154, H155, Y157, M158 in helix 2, and Y216 in helix 3 formed a “portaledge” which may be indispensable for heme binding and efflux. Meanwhile, Y291 in the bottom of TMD1 also affects the heme transport activity of CG4562/dMRP5, likely due to its role in maintaining structural stability as it is located between helix 4 and helix 5. Another possible explanation is that Y291 at the bottom of TMD1 may function to recruit labile heme in the cytosol. K346 and Y359 mutations also block the heme-transporting activity which may act as the exit of heme (Fig. 6B).

Unlike the residues in TMD1, the functional amino acids (H845, Y916, K924, Y935, H968, Y973) in TMD2 disperse from bottom to middle of the cavity. Together with Y291, K346, and Y359 in TMD1, we assume that these residues in TMD2 work as “pitons” for heme efflux from the cytosol to the extracellular space (Fig. 6C). All the affected mutants do not have localization changes (Additional file 1: Fig. S4G). The potential heme-binding residues of TMDs are shown in Additional file 1: Fig. S4D.

The bottom-view of CG4562/dMRP5 presents the classical “head-to-tail” configuration of NBD dimer as reported in all ABC transporters (Fig. 6D). The hallmark of the ABCC subfamily is the replacement of the active glutamate residue of the Walker B motif in the NBD1 domain with aspartate, which maintains the ATP binding activity but loses the ATP hydrolysis activity (Fig. 6E). Thus, the CG4562/dMRP5 NBD dimer formed two ATP binding sites, namely the consensus site and the degenerate site. A close-up view of the consensus site showed that the signature motif LSGGQ of the NBD1 domain and the Walker A motif, Q loop, Walker B motif, H loop, and A loop from the NBD2 domain formed the consensus site which binds and hydrolyses ATP, offering energy for conformation change and cargo transport. We have generated the consensus sites’ variants and validated that these variants lack the heme transporting activity (Fig. 6F). Meanwhile, a zoom-in view indicated that the signature motif FSVGQ of the NBD2 domain and the Walker A motif, Q loop, Walker B motif, and H loop from the NBD1 domain enclosed and formed the degenerate site. Mutants of representative residues in the degenerative site failed to export heme (Fig. 6G). Through amino acid sequence alignment and structure information, we also selected two “A loop” candidate sites of NBD1 (W452 and T460) and constructed the corresponding mutants. However, mutations in these two sites did not affect heme exporting activity, indicating that there may exist a noncanonical motif that functions like the A loop (Additional file 1: Fig. S4F).

Vanadate shares a similar structure to that of ATP, which could be used to block ATP-dependent transporters. Vanadate treatment significantly blocked the transport activity of CG4562/dMRP5. Noteworthy is that the vanadate treatment also somewhat reduced ZnMP fluorescence signal in the control, implying that some other ATP-dependent transporter might function in the basal ZnMP absorption (Additional file 1: Fig. S4C).

**Fig. 7:**
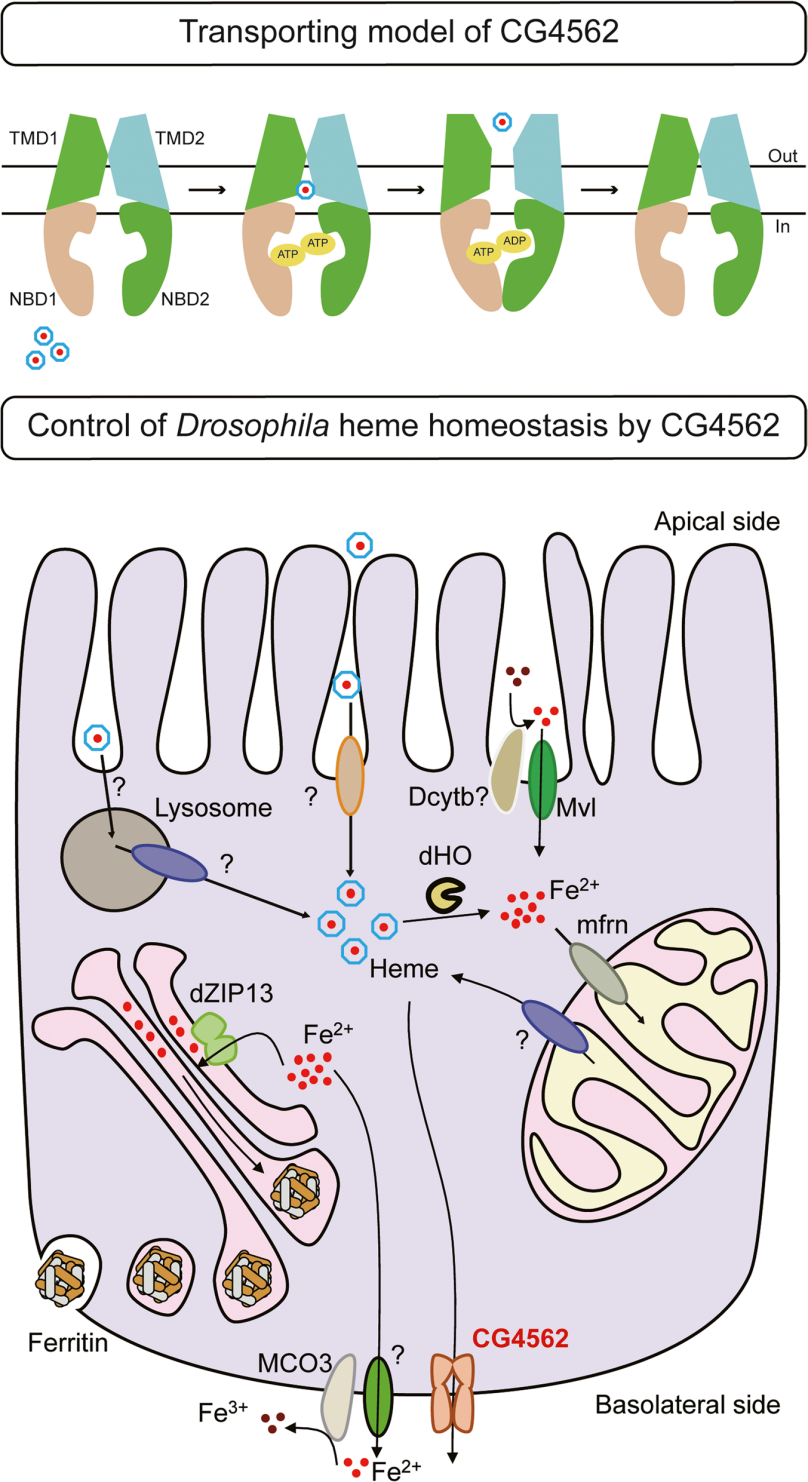
Transport mechanism and physiological function of CG4562/dMRP5. CG4562/dMRP5, which is a typical ABC transporter, facilitates heme transport out of cells with ATP hydrolysis. Heme, as an alternative iron source, is absorbed from outside of cells or synthesized in the mitochondria of the flies. Excess heme in the gut cells is degraded by heme oxygenase or detoxifies/effluxes by CG4562/dMRP5 located on the basolateral side of the enterocyte

In sum, with these biochemistry assays, we propose the working model of how heme is transported by CG4562/dMRP5: the active sites in TMD domains bind and stabilize heme in the cavity among the TMD domains, while NBD1 and NBD2 form two ATP-binding sites wherein some consensus sites hydrolyze ATP and offer energy for the conformation change of TMD helixes, releasing heme out to the extracellular space (Fig. 7).

## Discussion

ZnMP has previously been used in mosquito cells to monitor heme transport [35]. In this work, we implemented a ZnMP screen system with some improvement to identify potential heme transporters in S2 cells. In that assay, transcriptional expression analyses were undertaken to reveal genes responsive to heme. Because heme transporter genes are not all transcriptionally regulated by heme, some may be regulated translationally or post-translationally, or conceivably some even may not respond to heme at all. On the other hand, transcriptionally responsive genes may not possess transporting activity. In our study, *CG11898* is transcriptionally regulated by exogenous heme in S2 cells but there is no heme transport activity associated with it. Vice versa, CG7627 could actively transport heme out of cells, but the mRNA level did not change under heme exposure. Therefore, the gain of function screen through overexpression is a functionally more relevant assay to verify heme transport activity.

CG4562/dMRP5 functions at the surface of the gut as a heme exporter, and knocking down this gene resulted in severe phenotypes in *D. melanogaster*. Ubiquitously knocking down *CG4562/dMRP5* caused embryotic lethality in *D. melanogaster*, while loss of expression in the gut only resulted in an eclosion decrease. We speculate that CG4562/dMRP5 may also function in some other tissues besides the intestine, although none of the more than 10 drivers we tested reproduced the lethality. Disruption of MRP5 in the worm and zebrafish both produced severe phenotypes [28]. However, *Mrp5* knockout mice are viable with no overt phenotypes [55]. One explanation for this discrepancy is that there might be another redundant MRP member in the mammal. Supporting this notion is that recently MRP5 and its closest homolog MRP9 were reported to play a concerted role in regulating male reproductive function [56]. Our studies uncovered two proteins of the MRP family which could transport heme out of cells. In *D. melanogaster*, a lower organism, the two proteins may share the same function but act in different parts of the body. For the human being, the regulation or responsive system could be more complex. It thus could be envisioned that in higher organisms some proteins may be functionally redundant to overcome the unexpected damage. Consistently, another protein FLVCR1a has been reported participating in exporting de novo synthesized heme from mammalian intestinal cells, and loss of FLVCR1a in the duodenum impaired the normal cell proliferation [57]. Interestingly, the *D. melanogaster* FLVCR homolog CG1358 failed to present heme export activity in our S2 cell assays, whereas the only one FLVCR homolog identified in *Leishmania* parasite has been shown to function as a porphyrin importer [58]. It could be speculated but worth testing in the future, whether CG1358, which is functionally similar to FLVCR2 rather than FLVCR1a, plays a role in heme import process.

We have to point out that although our screen assay is robust in detecting heme export out of the cells, it would not be so in identifying proteins with intracellular heme transport ability. Therefore, while we identified two genes with heme exporting property, we cannot state that the others do not possess heme transporting activity. Having said that, adapting this system for importing assay would be possible. It remains to be seen whether there exists an HRG-1 like or plasma membrane-located transporters functioning for heme absorption in the flies.

## Conclusions

In this work, we embarked on a systemic characterization of heme transport in the model organism *D. melanogaster* and expanded our research to potential heme transporters in *A. aegypti*. Our results suggest that the heme homeostasis scheme in *D. melanogaster* appears overall resembling that of mammals. Some conserved proteins between mammals and *D. melanogaster* shared similar functions. Through analysis of a homology-modeled structure of CG4562/dMRP5, we provided some insights into the ATP binding pockets and the transport path during this heme excretion process. Collectively, *D. melanogaster* as a model organism may shed light not only in mammalian studies but also in pests, and heme metabolism studies in insects may offer potential new targets for pests’ control in the future.

Altogether, our findings characterized some potential heme transporters in *D. melanogaster* and *A. aegypti*. In mammals, possibly due to the high demand for heme, a complex heme regulation system has evolved. In *D. melanogaster*, the heme transport process appears to be more ancient. Nevertheless, some prototypes of heme homeostasis schemes might be similar in insects and mammals. We hope that our heme metabolism studies in *D. melanogaster* will complement existing heme research.

## Methods

### Plasmids

The *D. melanogaster* and *A. aegypti* candidate heme transporter genes were generated by PCR amplification of the coding region from *D. melanogaster* or *A. aegypti* cDNA. ORFs of these proteins were in-frame fused with C-terminal GFP tags and cloned into S2 cell expression vector pAC5.1. CG4562 was cloned into the *Drosophila* expression vector pUAST-attB, and UAS-hMRP5 was generated by PCR amplification of a plasmid from Iqbal Hamza laboratory and cloned into pUAST. Transgenic flies were made by microinjection into embryos with a vasa-driven phiC31 transgene and an attP insertion at the attB site (a gift from Dr. Guanjun Gao, School of Life Science and Technology, ShanghaiTech University) or *w*^*1118*^ background flies. Mutation of the genes was generated by overlapping PCR. All the constructs were verified by sequencing.

### Fly stocks, culture media, and transgenic flies

Fly stocks were raised at 18 °C, and all the experiments were performed at 25 °C (12 h daytime and 12 h nighttime, 60% humidity) on standard cornmeal food. If necessary, the food was supplemented with other chemicals. Fly stocks *Actin-GAL4/CyO* (Bloomington #4414), *Da-GAL4* (Bloomington #8641), and UAS-EGFP (Bloomington#5130) were obtained from the Bloomington Stock Center (Bloomington, IN, USA); *NP3084-GAL4* (DGRC# 113094) was from the *Drosophila* Genetic Resource Center at the Kyoto Institute of Technology (Kyoto, Japan). *Esg-GAL4*, *pros-GAL4*, and *myo1A-GAL4* are a gift from Wei Zhang lab (Tsinghua University, China). The RNAi lines used for the screen were attached in the supplement Additional file 1: Table S1.

Hemin was dissolved in DMSO at 100 mM. 4,6-Dioxoheptanoic acid (succinylacetone, SA) was dissolved in H_2_O at 50 mg/ml, and the zinc-mesoporphyrin (Logan, UT) was prepared in DMSO at 100 mM.

### Transfection and ZnMP assay

Vector transfections were performed by Effectene (Qiagen, German) following the standard transfection protocol with cells at 70–80% confluency. Specifically, 25 μl Buffer EC, 1 μg of the specific vector, 2 μl enhancer, and 2 μl Effectene were used for each 24-well plate. A control CD8 with a C-terminal GFP tag was performed with each experiment. At 2 days post-transfection, the cells were incubated with 5 μM ZnMP for 15 min at room temperature. The ZnMP and the GFP signal were analyzed by cytometry.

### Fly survival assay

GAL4 homozygous flies were crossed with different transgenic flies, and the progeny were reared on different diets, as indicated in each experiment. The density of each vial was controlled to 50 larvae, and the total number of emerged adults of each genotype was counted.

### RNA isolation, semi-quantitative RT-PCR, and quantitative real-time PCR

Total RNA was extracted from adults, or 3rd instar larvae using the Trizol reagent (Invitrogen, Carlsbad, CA, USA). cDNA was reverse-transcribed from 1 μg total RNA with TransScript First-Strand cDNA Synthesis SuperMix (TransGen Biotech Co., Ltd., Beijing, China), under the manufacturer’s instructions.

Real-time PCR was performed on CFX96 Touch Real-Time PCR System (Bio-Rad) using SYBR Green PCR Master Mix (TransGen Biotech Co., Ltd., Beijing, China). Fold change was determined by comparing target gene expression with the reference gene expression (*rp49*) in *Drosophila* and *AAEL-Actin* in *A. aegypti*. The primers used for real-time PCR were attached in the supplement Additional file 1: Table S2 and Additional file 1: Table S3.

### Immunofluorescence, ZnMP staining, and microscopy

For immunofluorescence examination of *Drosophila*, 3rd instar larvae were collected and dissected in phosphate-buffered saline (PBS), fixed with paraformaldehyde. For nuclear staining, samples were incubated in 50 ng/mL DAPI for 10 min and washed three times in PBS. Slides were mounted with 50% glycerol/PBS. ZnMP staining for flies is similar to immunofluorescence except for the sample treatment. Third instar larvae were reared on 100 μM ZnMP food for 1 day before dissection, and the following steps were the same as in the immunofluorescence analysis.

For immunofluorescence examination of cells, transfected cells were fixed with 10% paraformaldehyde, incubated in 5% Triton PBS buffer for 10 min, and nuclei DAPI stained. Slides were mounted with 50% glycerol/PBS for confocal picturing observed by Olympus FV3000.

### Total cellular heme quantification

Twenty 3rd instar larval guts were dissected, weighed, and homogenized. Hemin was extracted from the aqueous sample homogenated using four volumes of the extraction solvent, which was made of four volumes of ethyl acetate and one volume of glacial acetic acid. The resulting phases were separated by spinning in a microcentrifuge for 10 s at the maximum speed. Ten microliters of the supernatant was injected into a Waters (Millford, MA) Acquity ultra-performance liquid chromatography (UPLC) system consisting of a binary solvent manager, sample manager, column heater, a photodiode array (PDA) detector, and an Acquity UPLC BEH C18, 1.7 μM, 2.1 × 100 mm column. The hemin peak was measured at an absorption maximum of 398 nm and quantified relative to a standard solution subjected to the same extraction method.

## Supplementary Information

**Additional file 1: Figure S1.** The phylogenetic tree of MRP family members in *D. melanogaster* and the *A. aegypti*, related to Fig. 1. **Figure S2.** Localizations of *Drosophila* MRPs in S2 cell and their expressions under hemin supplementation in S2 or Aag2 cells. **Figure S3.** Phenotypic analysis of CG4562 and MRPs expression level in adults’ intestine, related to Fig. 5. **Figure S4.** Some residues of CG4562/dMRP5 could be mutated without influencing the transport activity, related to Fig. 6. **Table S1.** RNAi List of *D. melanogaster.* **Table S2.** qPCR Primers of *D. melanogaster.* **Table S3.** qPCR Primers of *A. aegypti.*

**Additional file 2: Movie S1.** ZnMP uptake by S2 cells at room temperature. **Movie S2.** ZnMP uptake by S2 cells at 4°C. **Movie S3.** ZnMP exported by CD8-GFP cell at room temperature. **Movie S4.** ZnMP exported by CG4562/dMRP5-GFP cell at room temperature.

**Additional file 3.** The individual raw data values of figures and Additional files for number of replicates ≤ 6. All the data are cited in the figure legend.

## Data Availability

All data generated or analyzed during this study are included in this published article and its supplementary information files.
