# Supplementary material for: Identification and characterization of a heme exporter from the MRP family in Drosophila melanogaster
Source: BMC Biol. 2022 Jun 2;20:126. doi: 10.1186/s12915-022-01332-0 (PMC9161523; doi:10.1186/s12915-022-01332-0)
Supplement: Supplementary file 1 — Additional file 1: Figure S1. The phylogenetic tree of MRP family members in D. melanogaster and the A. aegypti, related to Fig. 1. Figure S2. Localizations of Drosophila MRPs in S2 cell and their expressions under hemin supplementation in S2 or Aag2 cells. Figure S3. Phenotypic analysis of CG4562 and MRPs expression level in adults’ intestine, related to Fig. 5. Figure S4. Some residues of CG4562/dMRP5 could be mutated without influencing the transport activity, related to Fig. 6. Table S1. RNAi List of D. melanogaster. Table S2. qPCR Primers of D. melanogaster. Table S3. qPCR Primers of A. aegypti. [file 12915_2022_1332_MOESM1_ESM.docx]

Figure S1. The phylogenetic tree of MRP family members in *D. melanogaster* and the *A. aegypti*, related to Fig 1.

**(A)** Heme synthesis homologous proteins in *Drosophila*. *Drosophila* genome harbors all corresponding heme synthesis gene homologs.

**(B)** The phenotype of knocking-down *dALAS* (*CG3017)* in the whole body by *Da-GAL4*. All values are available in Additional file 3: Fig S1B.

**(C)** Phylogenetic tree illustrating relationships among human and putative *Drosophila* MRP genes. The tree was generated and displayed with the ClustalX and MEGA software.

**(D)** Phylogenetic tree illustrating relationships among *Drosophila* and putative *A. aegypti* MRP4 orthologue genes. The tree was generated and displayed with ClustalX and MEGA software.

Data are presented as the mean ± SD by two-tailed unpaired Student’s t-test.. *p < 0.05, **p < 0.01; ***p < 0.001. Experiments were repeated at lease two times, and data from only one representative experiment were shown.

**
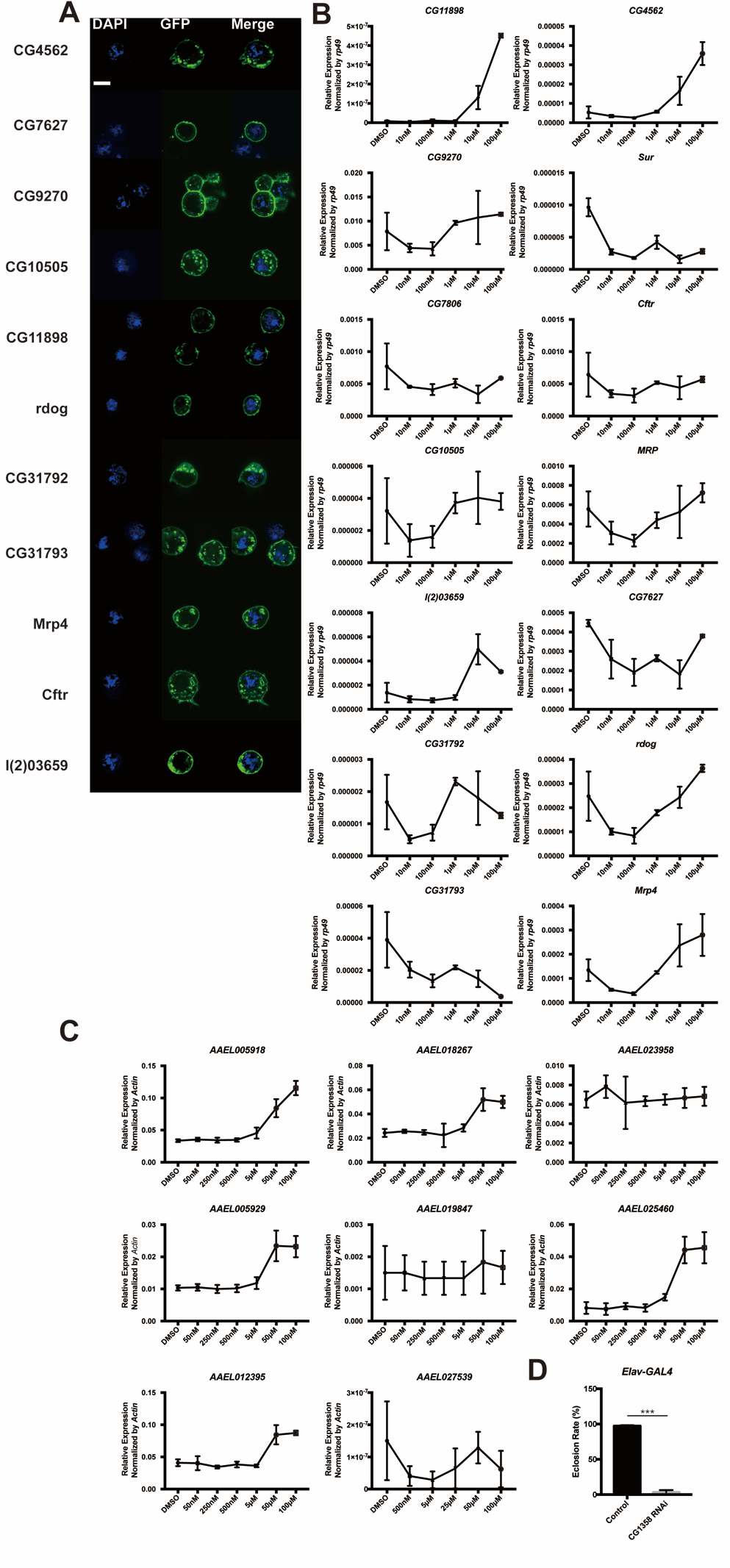
**

Figure S2. Localizations of *Drosophila* MRPs in S2 cell and their expressions under hemin supplementation in S2 or Aag2 cells.

**(A)** *Drosophila* MRPs were expressed in S2 cell and their subcellular localizations were examined with confocal microscopy.

**(B)** Expressions of MRPs in S2 cells when treated with different concentrations of hemin. Expressions were quantified by real-time relative RT-PCR normalized to *rp49*. All values are available in Additional file 3: Fig S2B.

**(C)** Expressions of MRPs in mosquito Aag2 cells under hemin. Expressions were quantified by real-time relative RT-PCR normalized to *AAEL-Actin*. All values are available in Additional file 3: Fig S2C.

**(D)** Phenotypic analysis of knocking down CG1358 by *Elav-GAL4.* All values are available in Additional file 3: Fig S2D.

Data are presented as means ± SD by two-tailed unpaired Student’s t-test. *p < 0.05, **p < 0.01; ***p < 0.001. Experiments were repeated at lease two times, and data from only one representative experiment were shown.


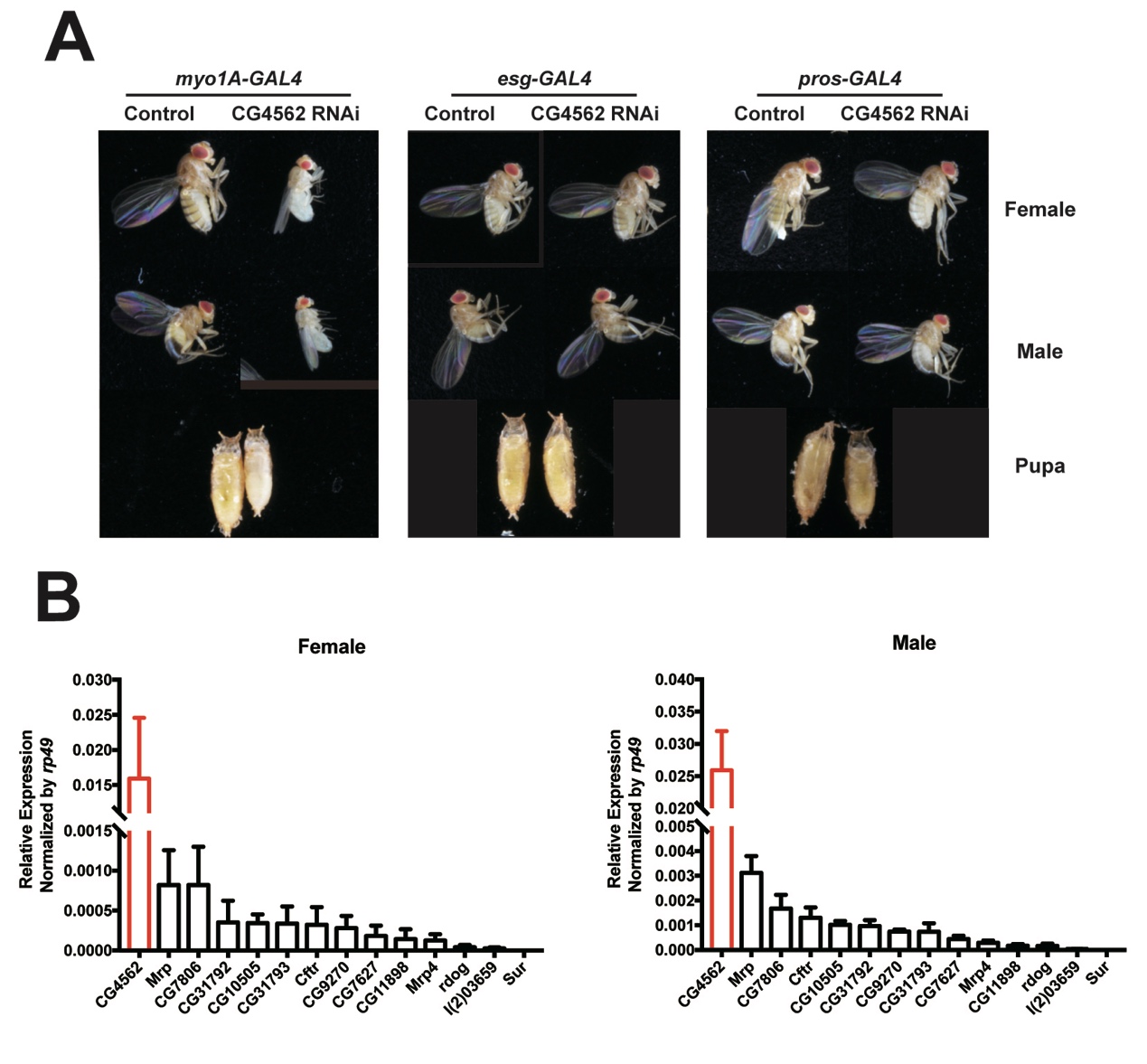
Figure S3. Phenotypic analysis of CG4562 and MRPs expression level in adults’ intestine, related to Fig 5.

**(A)** Flies and pupae after *CG4562* knockdown by different gut GAL4s. *Esg-GAL4* expresses in the ISCs, *myo1A-GAL4* expresses in the ECs, and *pros-GAL4* expresses in the EEs.

**(B)** Relative expression levels of MRPs in female and male intestine. All values are available in Additional file 3: Fig S3B.

Data are presented as means ± SD. Experiments were repeated at lease two times, and data from only one representative experiment were shown.

**
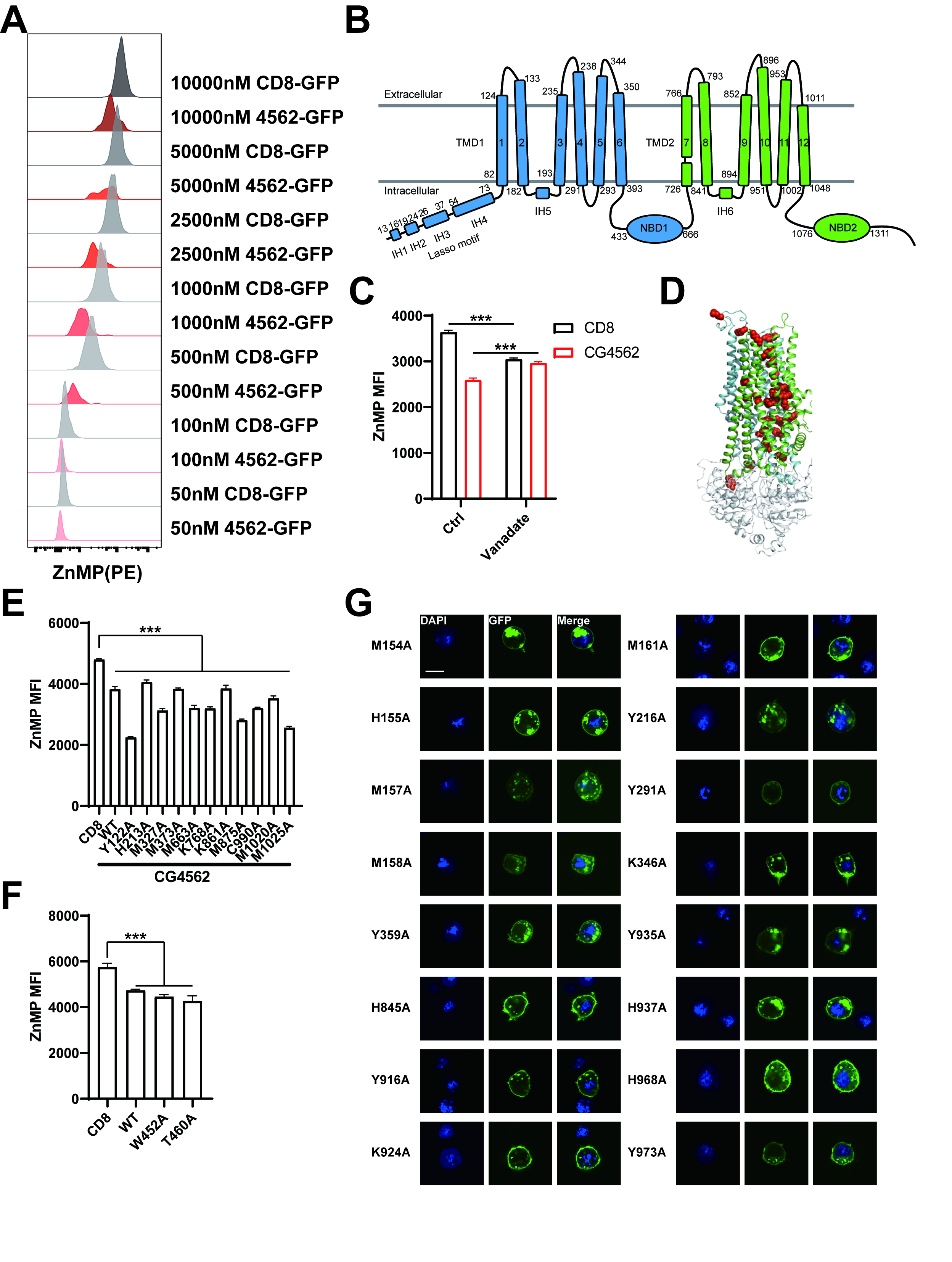
**

Figure S4. Some residues of CG4562/dMRP5 could be mutated without influencing the transport activity, related to Figure 6.

**(A)** Heme export activity of CG4562/dMRP5 was analyzed in transfected S2 cells incubated with various concentrations of ZnMP.

**(B)** Topological diagram of CG4562/dMRP5.TMD, transmembrane domain; NBD, nucleotide-binding domain; IH, intracellular helix.

**(C)** Effect of vanadate on the fluorescence of ZnMP of S2 cells over-expressing CG4562/dMRP5. All values are available in Additional file 3: Fig S4C.

**(D)** Topological diagram of CG4562/dMRP5. The red spots represent important residues of the transmembrane domains for CG4562/dMRP5.

**(E)** Quantitative measurement of ZnMP fluorescence of CG4562/dMRP5 mutants with non-critical TMD mutations. All values are available in Additional file 3: Fig S4E.

**(F)** Quantitative measurement of ZnMP fluorescence of CG4562/dMRP5 mutants with non-critical NBD mutations. All values are available in Additional file 3: Fig S4F.

**(G)** Localization of CG4562/dMRP5 mutant proteins with critical transmembrane residue changes when expressed in S2 cells. Scale bar = 10 nm

Data are presented as the mean ± SD by two-tailed unpaired Student’s t-test except for (C) by one-way ANOVA test. *p < 0.05, **p < 0.01; ***p < 0.001. Experiments were repeated at lease two times, and data from only one representative experiment were shown.

**TABLE S1 RNAi List of *D. melanogaster***

| Gene name | Num. | Ubiquitous RNAi  (*Actin-GAL4*) | Gut RNAi  (*NP3084-GAL4*) |
| --- | --- | --- | --- |
| MRP RNAi screen | | | |
| *CG7627* | THU0793 (THFC) | Lethal | Normal |
| *CG7627* | V#101084 (VDRC) | Lethal | Normal |
| *CG9270* | THU1382 (THFC) | Normal | Normal |
| *CG9270* | V#29961 (VDRC) | Normal | Normal |
| *CG9270* | V#109785 (VDRC) | Normal | Normal |
| *CG10505* | THU3822 (THFC) | Normal | Normal |
| *CG10505* | V#107842 (VDRC) | Normal | Normal |
| *CG11898* | TH04030.N (THFC) | Normal | Normal |
| *CG11898* | V#100660 (VDRC) | Normal | Normal |
| *CG11898* | B#64599 (BDSC) | Normal | Normal |
| *l(2)03659* | TH04308.N (THFC) | Normal | Normal |
| *l(2)03659* | V#39540 (VDRC) | Normal | Normal |
| *l(2)03659* | B#67292 (BDSC) | Normal | Normal |
| *Mrp4* | TH04314.N (THFC) | Normal | Normal |
| *Mrp4* | B#60136 (BDSC) | Normal | Normal |
| *Mrp4* | V#101221 (VDRC) | Normal | Normal |
| *Mrp4* | V#6053 (VDRC) | Normal | Normal |
| *CG31792* | B#34942 (BDSC) | Normal | Normal |
| *CG31792* | V#107237 (VDRC) | Normal | Normal |
| *CG31793* | B#38319 (BDSC) | Normal | Normal |
| *Cftr* | B#57570 (BDSC) | Normal | Normal |
| *Cftr* | V#1204 (VDRC) | Normal | Normal |
| *rdog* | V#28259 (VDRC) | Normal | Normal |
| *rdog* | B#38318 (BDSC) | Normal | Normal |
| *rdog* | B#68002 (BDSC) | Normal | Normal |
| *CG4562* | THU1025 (THFC) | Lethal | Lethal |
| *CG4562* | 4562R-1 (NIG) | Lethal | Lethal |
| *CG4562* | 4562R-2 (NIG) | Normal | Normal |
| *CG4562* | V#6770 (VDRC) | Normal | Normal |
| *CG4562* | V#106975 (VDRC) | Normal | Normal |
| Non-MRP RNAi screen | | | |
| *CG3156* | TH04223.N (THFC) | Normal | Normal |
| *CG3156* | B#57727 (BDSC) | Normal | Normal |
| *Hmt-1* | TH02405.N (THFC) | Normal | Normal |
| *Hmt-1* | B#53284 (BDSC) | Normal | Normal |
| *w* | THU0558 (THFC) | White eye | Pink eye |
| *w* | THU0583 (THFC) | Normal | Normal |
| *CG30345* | THU1348 (THFC) | Normal | Normal |
| *CG30345* | V#103652 (VDRC) | Normal | Normal |
| *CG1358* | V#101453 (VDRC) | Partial lethal | Normal |
| *CG1358* | 1358R-1 (NIG) | Lethal | Normal |
| *CG1358* | 1358R-2 (NIG) | Lethal | Normal |
| *CG1358* | V#13313 (VDRC) | Lethal | Normal |

Abbreviation: VDRC (Vienna Drosophila RNAi Center, Austria), BDSC (Bloomington Drosophila Stock Center, America), NIG (Fly Stock of National Institute of Genetics, Japan) and THFC (Tsinghua Fly Center, China)

**TABLE S2 qPCR Primers of *D. melanogaster***

| Gene | Forward Primer | Reverse Primer |
| --- | --- | --- |
| *rp49* | CCGCTTCAAGGGACAGTATCTG | ATCTCGCCGCAGTAAACGC |
| *CG3017* | TTCGGGTAACTCTCTTCACCAC | ATCTTGGGCACGCTTTTGTC |
| *CG14716* | TGCTGCCCAAAGAATTCCAC | AGCAAGAGTTCGTTCTGTGC |
| *Cftr* | GGCGATTCTGCGAAACAACA | GGCGACACCAGCATCCATTA |
| *CG4562* | GAGGGGCGCATAGGAATGAA | CGCGTCTCAATCTGGCCATA |
| *CG7627* | CGATGTGGGACTGGCCATTA | CGATTTTTCCCTGCTCTGGC |
| *CG7806* | GCGTATTGGCATCGTTGGAC | CAGATTTTCCCGCACAGTGC |
| *CG9270* | AATTCCCCAGGAACCTGTGC | CAGACCAACTGCCTCTGACC |
| *CG10505* | GGTGGCTCCAACTTCAGCAT | CAGTGTGCAATCTGTGAGCG |
| *CG11898* | AACTTCAGCATGGGCCAGAG | ACCCGGTCGTTGTCCATAAC |
| *CG31792* | TCATGGACTCGGACCGTGTA | GCATAAGGCTCGGTTTTTCAGA |
| *CG31793* | CGCAGTGGTGGTCAAATTGG | ACAAATGACCAGGCCCACAT |
| *l(2)03659* | TTCTTTGCCTTTCCTCCGGG | CTTCTTGTCGTCCGGAGCTT |
| *MRP* | CAGAACTTCCAGGTGCGCTA | TGCAGACCCATTGAGGCAAT |
| *Mrp4* | TCTGGGGCTGTACAAGGAGT | CATTGAGCGCGGCAAAGTAA |
| *Sur* | AACGGATTGGCATCTGTGGT | TTCACGTATGGTCGCGTTGA |
| *rdog* | TCTGGGCATGATACGCTACC | AAGGAAGAGAAACCAGGCGG |

**TABLE S3 qPCR Primers of *A. aegypti***

| Gene | Forward Primer | Reverse Primer |
| --- | --- | --- |
| AAEL005918 | TTCGGTATGATAAGGGCCATC | GGTTAGCACCGATTGTGTGG |
| AAEL025460 | CGATCATCCCTCAAGATCCG | CTGGATCAAACGATCGGTATTTGG |
| AAEL012395 | ACGTGGATCCAGAAACCGAC | GCAGATATCCACTGGGACCG |
| AAEL023958 | GGAGGACTCGATGCGAAGAT | AACCAGTTTGTCGGTTTCTGGAT |
| AAEL027539 | GCGGCACTTGAGCAGGTAAAAC | ATCAGGATCCGACTGTCCCG |
| AAEL018267 | GTTGAAGCCACGACACGCTC | ATGGCCGTCGAATTCACTCA |
| AAEL019847 | CTTTGGCCGGAGGACTCAAT | GGATGAGATCGTCGGTTTCTGA |
| AAEL-Actin | GAACACCCAGTCCTGCTGACA | TGCGTCATCTTCTCACGGTTAG |
